# Pressurized Liquid Extraction (PLE) as an Innovative Green Technology for the Effective Enrichment of Galician Algae Extracts with High Quality Fatty Acids and Antimicrobial and Antioxidant Properties

**DOI:** 10.3390/md16050156

**Published:** 2018-05-10

**Authors:** Paz Otero, Somaris E. Quintana, Guillermo Reglero, Tiziana Fornari, Mónica R. García-Risco

**Affiliations:** Research Institute of Food Science (CSIC-UAM). C/Nicolás Cabrera 9, Autonomous University of Madrid, 28049 Madrid, Spain; somaris.quintana@predoc.uam.es (S.E.Q.); guillermo.reglero@uam.es (G.R.); tiziana.fornari@uam.es (T.F.); monica.rodriguez@uam.es (M.R.G.-R.)

**Keywords:** macroalgae, *Fucus vesiculosus*, fatty acids, pressurized liquid extraction (PLE), gas chromatography-mass spectrometry (GC-MS)

## Abstract

Marine organisms are potentially prolific sources of high qualify fatty acids that represent useful leads in the development of new nutraceutical agents. In this work, we investigated the lipid composition of six algae species from the Northwest of Spain (*Ulva intestinalis*, *Ulva lactuca*, *Fucus vesiculosus,* *Dictyota dichotoma, Cystoseira baccata* and *Himanthalia elongata*) and compared the antioxidant and antibacterial activity of ethanolic extracts obtained by pressurized liquid extraction (PLE). Furthermore, *Fucus vesiculosus* (*F. vesiculosus)* PLE using five solvents of different polarities (hexane, ethyl acetate, acetone, ethanol and ethanol:water 50:50) at three temperatures (80 °C, 120 °C and 160 °C) was investigated. *F. vesiculosus* ethanolic PLE extract presents considerably higher capacity of inhibiting 50% of DPPH (1,1-diphenyl-2-picryl hydrazyl) (IC_50_ = 7.17 μg/mL) in comparison with the rest of macroalgae studied. Moreover, the potential antimicrobial activity tested on *E. coli* and *S. aureus* shows that *F. vesiculosus* extract produced the best inhibition (IC_50_ was 2.24 mg/mL (*E. coli*) and 1.27 mg/mL *(S. aureus*)). Furthermore, regarding the different solvents and temperatures used to investigate *F. vesiculosus* PLE, results showed that this technique using ethyl acetate is a selective method to enrich long chain fatty acids (oleic acid, arachidonic acid and eicosapentaenoic acid) with ω-6/ω-3 ratios close to 2.7.

## 1. Introduction

The economic potential of the algae industry is widely recognized. Algae have been consumed for many years in Asian countries and their bioactives have recently gained a considerable interest due to their multiple applications in the food and pharmaceutical industries [1,2,3]. Two major types of marine algae can be identified: the macroalgae or seaweeds occuping the littoral zone, which included green algae (Chlorophyceae), brown algae (Phaeophyceae) and red algae (Rhodophyceae), and the microalgae found in both benthic and littoral habitats and also throughout the ocean waters as phytoplankton [4].

Although algae consumption around the world has increased considerably mainly due to the Asian influence and its cuisine [5], in most developed countries, people dislike eating algae directly as food commodity due to their dirty image and the lack of reliability in regards to non-toxic products. Extracting algae compounds and using them as food ingredients increase consumer acceptance. Over the past decades, bioactive properties of algae components have been investigated and several algae extracts have been brought to the market as food supplements. For example, Solaray^®^, a dietary supplement product containing the extract of the red marine algae *Rhodymenia palmata* is sold to maintain the health of the immune system; Omega 3 Vegan Lineavi, a product from *Schizochytrium* sp. algae oil, provides a vegetarian and vegan friendly source of the healthy fats [6]. Omega-3 fatty acids are mainly found in unicellular phytoplankton and marine algae. Eicosapentaenoic acid (EPA) accumulates in fish and other marine animals that consume algae and get passed on to other species through the food chain [7,8]. These FAs have been found to have positive effects on the central nervous system for the development of brain, retinal and neural tissues in foetuses and young children [8]. Hence, the importance of algae as a source for high value FAs intended for nutrition is increasing rapidly and methods for maximum extraction and best determination of different fatty acid (FAs) in algae are needed.

Traditionally these lipids have been extracted using Solid–Liquid Extraction (SLE) methods based in chloroform:methanol in different proportions [9,10], and subsequent FA profiling is carried out by LC-MS or GC-MS. Recently, acid-based extraction was used for improving the lipid yield of lipid-producing microalgae, being hypochlorous and sulphuric acids the most commonly used in an acid-catalysed hot-water extraction [11,12,13,14]. However, SLE is time-consuming, energy intensive and not in accordance with the current regulation about the use of permitted solvents in the production of foodstuffs and food ingredients [15]. Therefore, it is necessary to identify and develop new efficient extraction processes to exploit the bioactives present in marine algae. Extraction technologies such as Supercritical Fluid Extraction (SFE) and Ultrasonic Solvent Extraction (USE) have been investigated to extract these FAs [16,17]. Moreover, PLE is currently considered an advanced technique since it offers important benefits such as shorter extraction time, decreased solvent consumption, decreased sample handling and increased yield [18]. Moreover, this technique is in line with the green aspects of sample preparation.

This work aims to study the FA content of macroalgae species originally from the Northwest of Spain, and to optimize the PLE technique for producing algae extracts with potential use as food supplements. In a first study, we compared the lipid content of four macroalgae Phaeophyta species, *Fucus vesiculosus* (*F. vesiculosus*)*, Dictyota dichotoma* (*D. dichotoma*)*, Cystoseira baccata* (*C. baccata*) and *Himanthalia elongata* (*H. elongata*) and two Chlorophyta species, *Ulva intestinalis* (*U. intestinalis*) and *Ulva lactuca* (*U. lactuca*). Moreover, ethanolic PLE extracts of these six macroalgae were produced and their antioxidant and antibacterial capacity were also compared. In a second study, the PLE optimization for the extraction of FAs was investigated in *F. vesiculosus*. To our knowledge, no assessment on the effect of PLE extraction on lipid composition of macroalgae has been done so far. While a number of researches optimized PLE to extract carotenoids and phenolic compounds from algae [19,20,21,22,23], none have been reported about the use of PLE to extract FAs from macroalgae.

## 2. Results and Discussion

### 2.1. Comparison of Macroalgae Lipid Content

The lipid content and FA profile of four brown species of macroalgae (*F. vesiculosus*, *D. dichotoma*, *C. baccata* and *H. elongata*) and two green algae (*U. intestinalis*, *U. lactuca*) were determined. Algae lipid fraction was extracted by Folch method and then, it was submitted to a trans-esterification step before being analysed by GC-MS. Table 1 shows the results obtained for the six macroalgae investigated. The lipid content ranged from 4.6% to 6.7% and, as expected, lower quantities were obtained for green algae. The six algae accumulated saturated FA (SFA) such as myristic (C14:0), palmitic (C16:0) and stearic (C18:0), but also unsaturated FA (USFA) like oleic (C18:1), linoleic (C18:2) and palmitoleic (C16:1). Besides, pentadecilic (C15:0) was detected in four species, γ-linolenic (C18:3) was identified in three species (*F. vesiculosus*, *H. elongata*, *U. lactuca*), ARA (C20:4) was detected in *F. vesiculosus* and *C. baccata* and EPA (C20:5) was detected in the *F. vesiculosus*, *C. baccata* and *D. dichotoma*.

The FA profiles found in the six alga species are in accordance with the profiles found in previous studies [2,24]. Schmid and Stengel studied the FA content (%) of *H. elongata* collected in the Irish Coast [24]. Besides palmitic acid (C16:0) (23.6%), the algae produce high content of ARA (C20:4) (16.6%) stearidonic acid (C18:4), γ-linolenic acid (C18:3) (10.7%), oleic acid (C18:1) (10.6%) and EPA (C20;5) (10.2%). Andrade and co-workers investigated the FA profile of algae species belonging to genus *Fucus*, *Cystoseira* and *Ulva* (*F. spiralis*, *C. tamariscifolia*, *C. usneoides*, *C. nodicaulis* and *U. lactuca*) from the West Coast of Portugal [2]. In general, FA profiles found in *Fucus* species are similar in both Portuguese and Galician algae species. The major fatty acid was oleic acid (C18:1) and the amounts were 21.69 mg/g (*F. spiralis*) and 13.15 mg/g (*F. vesiculosus*). Similarly, the major FA found in *Ulva* and Cystoseira species was palmitic acid (C16:0) and the amounts were 0.38 mg/g (*U. lactuca* Portuguese specie), 6.09 mg/g (*U. lactuca* Galician specie), 6.02 mg/g (*U. intestinalis*), 6.80 mg/g (*C. baccata*), 0.29 mg/g (*C. tamariscifolia*), 0.20 mg/g (*C. usneoides*) and 0.09 mg/g (*C. nodicaulis*).

### 2.2. Comparison of Antioxidant and Antibacterial Activities of Ethanolic PLE Macroalgae Extracts

The antioxidant and antimicrobial activity of these macroalgae were also studied and compared. For this purpose, ethanolic PLE extracts were obtained at 120 °C. Antioxidant capacity was tested using the DPPH assay and results are shown in Table 2. The best dose-response activity was observed for *F. vesiculosus* followed by *C. baccata* and *H. elongata* with a capacity of inhibiting 50% of DPPH (IC_50_) at 7.17 ± 0.01 μg/mL (*F. vesiculosus*), 28.49 ± 3.80 μg/mL (*C. baccata*) and 64.89 ± 6.64 μg/mL (*H. elongata*). Our results concord with previous studies which describe green algae have lower free radical scavenging activities when compared to brown algae [25,26,27]. While high IC_50_ values are found for pure or aqueous methanol extracts from *Ulva* species such as *U. clathrata* (715 μg/mL) [28], *U. prolifera* (3026 μg/mL) [28] and *U. compresa* (>1000 μg/mL) [26], low IC_50_ values are observed in pure or aqueous methanol/ethanol extracts from *Cystoseira* species like *C. sedoides* (27 μg/mL) [29], *C. baccata* (28 μg/mL), *C. usneoides* (55 μg/mL) [26], *C. osmundacea* (69 μg/mL) [27] and *C. tamariscifolia* (109 μg/mL) [26]. At the same time, the genus *Fucus* is more reactive than *Cystoseira* after being extracted with organic solvents. The IC_50_ values obtained by DPPH assay are about 5 μg/mL for *F. distichus* [30], 7 μg/mL for *F. vesiculosus* (from Galician Coast), 10–14 μg/mL for *F. vesiculosus* (from French Coast) [31] and 178 μg/mL for *F. spiralis* [26]. Data regarding genus *Himanthalia* and *Dyctiota* are scarce in the literature. A recent study shows that despite *D. dichotoma* extracts does not produced any antioxidant activity by the DPPH assay, interestingly this alga has the higher anti-cancer activity out of 24 marine macroalga studied [32]. To conclude, results of the DPPH assay show large variation in free radical scavenging activity among the different macroalgae genera. However, the activity of the *Fucus* and *Cystoseira* extracts obtained by PLE in this study are comparable to those obtained by SLE described and referenced above.

**Figure 1:**
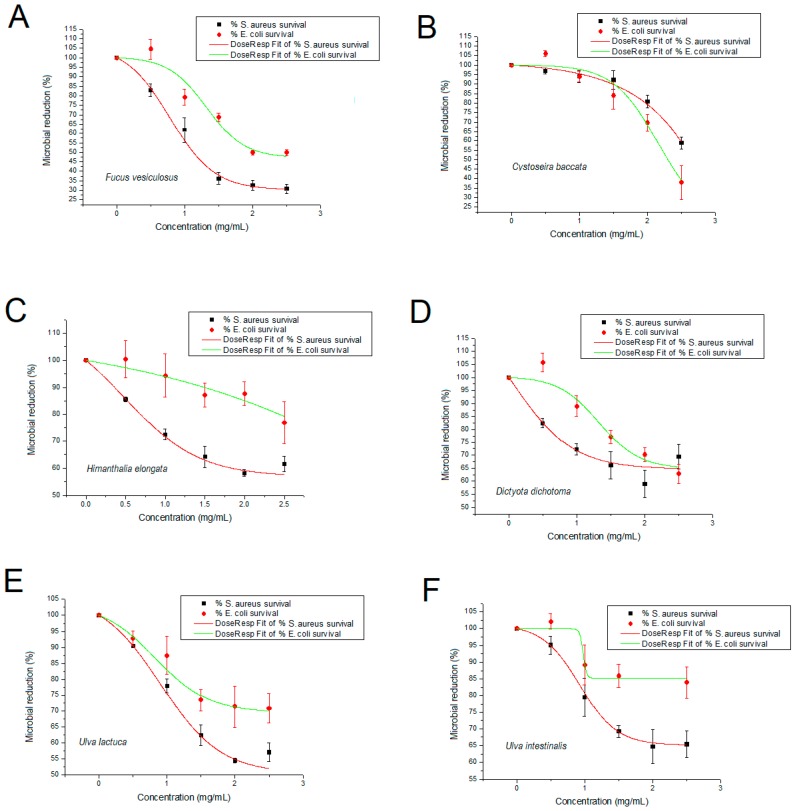
Antimicrobial activity of the PLE ethanoic extracts seaweeds against the two pathogenic bacterial strains. The activity is reported as % of antimicrobial reduction against the controls and each graph correspond to *Fucus vesiculosus* (**A**), *Cystoseira baccata* (**B**), *Himanthalia elongata* (**C**), *Dictyota dichotoma* (**D**), *Ulva lactuca* (**E**) and *Ulva intestinalis* (**F**) PLE extracts. Results show mean ± standard error of the mean (SEM) of three experiments.

The antimicrobial activity of the six extracts from Spanish species were tested for a Gram-negative bacteria, *Escherichia coli* (*E. coli*) and a Gram-positive bacteria, *Staphylococcus aureus* (*S.aureus*). Figure 1 shows the activity reported as % of antimicrobial reduction against the controls. Despite the six species (Figure 1A–F) showed antimicrobial activity against tested organisms, *F. vesiculosus* (Figure 1A) followed by *C. baccata* (Figure 1B) showed the best results. *F. vesiculosus* extract (2.5 mg/mL), inhibited the grown of *S. aureus* and *E. coli* in 30.7% and 49.8%, respectively. In parallel, *C. baccata* extract (2.5 mg/mL), inhibited the grown of the same pathogens in 58.8% (*S. aureus*) and 37.9% (*E. coli*) after 24 h.

**Figure 2:**
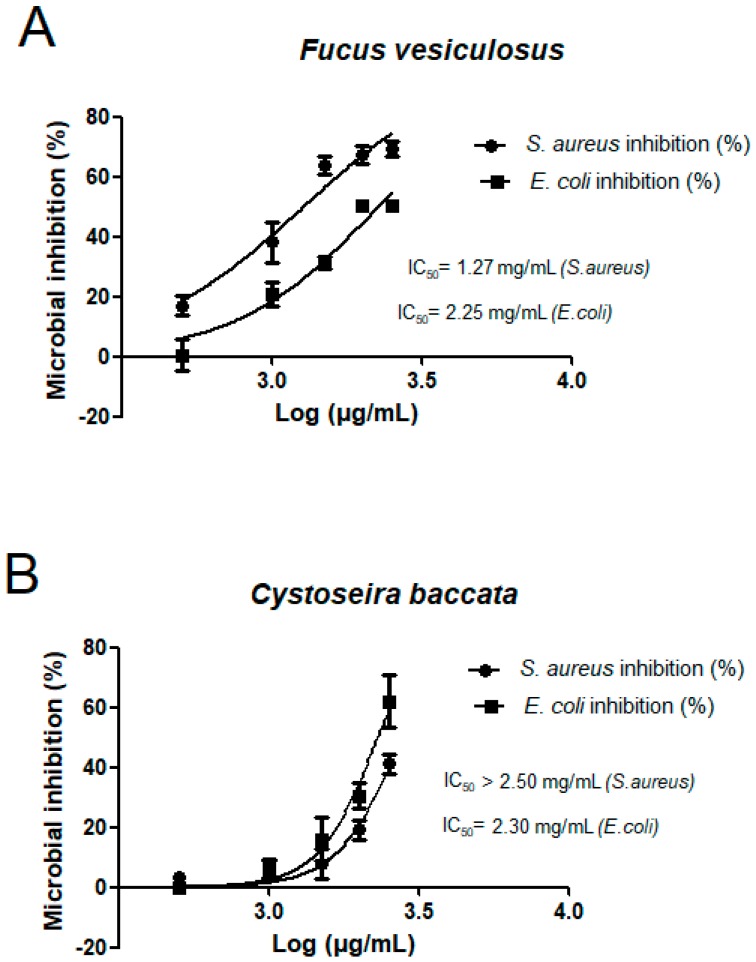
Antimicrobial activity of the PLE ethanoic extracts *F.vesiculosus* (**A**) and *C. baccata* (**B**) against the two pathogenic bacterial strains. Results show mean ± standard error of the mean (SEM) of three experiments.

For these two algae, the inhibition concentration (IC_50_) values were calculated and results are shown in Figure 2. *F. vesiculosus* produced the best inhibition, IC_50_ was 2.24 mg/mL (*E. coli*) and 1.27 mg/mL (*S. aureus*). Despite preliminary results showed that different species of algae have an antimicrobial potential especially against *E. coli* and *S. aureus* [33,34,35], there are no many studies about the antibacterial activity of seaweeds used in the present study. El-Amraoui and co-workers evaluated the antimicrobial activity of several macroalgae extracts from the Maroccan Atlantic Coast [36]. They also found that the brown algae belonging to genus *Cystoseira and Fucus* (*C. brachycarpa*, *C. compressa*, *F. vesiculosus*) together with the red alga *Gelidium sesquipedale* had the best antimicrobial activity out of ten tested species (SLE with ethanol). Gupta et al. [37] evaluated the antibacterial activity of the edible Irish brown seaweeds *H. elongata*, *Saccharina latissimi* and *Laminaria digitata*. Their activity was tested against pathogens which commonly cause problems in the food industry, *Listeria monocytogenes*, *Salmonella abony*, *Enterococcus faecalis* and *Pseudomonas aeruginosa*. Methanol extracts of raw *H. elongata* (60 mg/mL) inhibited *Listeria moncytogenes* by 98.7% compared to 96.5% inhibition by the synthetic preservative standard sodium benzoate and sodium nitrite (96.2%). In our study, we used PLE extracts of *H. elongata* 24 times less concentrated (2.5 mg/mL) and they inhibited 38% *S. aureus* growth and 22% *E. coli* growth. Previous studies have demonstrated that algae can produce a wide variety of metabolites as defences against herbivory as well as to prevent biofouling [38]. The substances isolated from macroalgae showing potent antimicrobial activity belong to polysaccharides, FA, phlorotannins, pigments, lectins, alkaloids, terpenoids and halogenated compounds [39,40], hence several compounds are involved in this activity including FA [33]. Moreover, the chemical composition of the algae and the antimicrobial activities vary with environmental conditions of the growth including light, temperature or salinity and geographical location and seasonality [40]. To summarize, the present finding brings out a new insight towards the possible development of antimicrobials against Gram-negative and Gram-positive bacteria based on *F. vesiculosus* and *C. baccata* natural products obtained by PLE green technology.

### 2.3. Effect of PLE on the Lipid Composition of F. vesiculosus Extract

According to the results of Section 2.1, *F. vesiculosus* accumulated the biggest number and quantities of FAs, thus the effect on the lipid profile of different PLE conditions was investigated using this alga. Parameters that have a significant effect on PLE efficiency were investigated, including solvent type and temperature. Precisely, 1 g of *F. vesiculosus* algae powder was submitted to PLE using five different solvents (hexane, ethanol, ethyl acetate, acetone and ethanol:water 50:50) and three temperatures (80 °C, 120 °C and 160 °C). The solvents used cover a wide range of dielectric constants, and therefore, able to extract bioactives of different polarity. After 10 min extraction, the resulting extracts were collected, dried and weight in order to determine the yield. Table 3 shows the yields obtained for each experiment. Comparing solvents, the higher amounts were obtained for the most polar ones (ethanol:water 50:50, ethanol and acetone), while low yields were achieved with ethyl acetate and hexane. For example, at 120 °C (intermediate extraction temperature tested) it was obtained 414.9 ± 14.7 mg (ethanol:water 50:50), 119.8 ± 11.8 mg (ethanol), 107.3 ± 2.3 mg (acetone), 55.9 ± 2.1 mg (ethyl acetate) and 37.2 ± 2.4 (hexane). As expected, the yield increased with the extraction temperature used in the PLE system [22], reaching the maximum amount at 160 °C with ethanol:water 50:50 (571.9 ± 23.8 mg).

The FA profiles of the *F. vesiculosus* PLE extracts were determined by GC-MS. In the chromatograms it was possible to identify up to eleven FA, including ten previously detected using Folch extraction and eicosa-5,8,11-trienoic acid (mead acid) (C 20:3) in small quantities at the end of the chromatogram (RT = 21.233).

**Figure 3:**
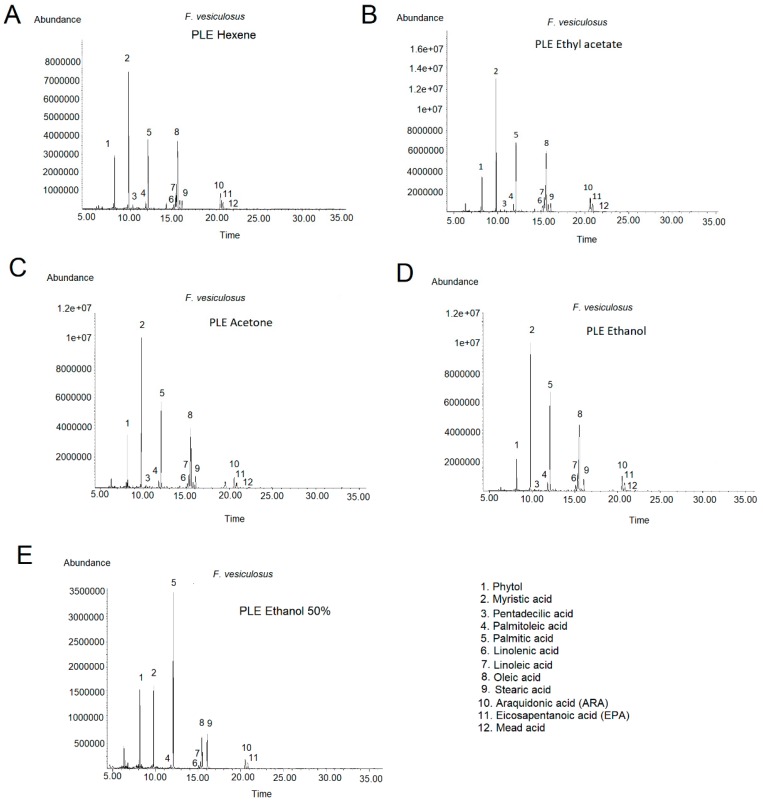
GC-MS chromatograms of *Fucus vesiculosus* extracted by PLE at 120 °C with hexane (**A**), ethyl acetate (**B**), acetone (**C**), ethanol (**D**) and ethanol:water 50:50 (**E**). The column used for FAMEs separation was an Agilent HP-5MS UI capillary column (30 m × 0.250 mm × 0.25 μm) and the oven temperature programme started at 50 °C, increased to 210°C at 20 °C increase per min and hold for 18 min. Then, temperature was further increased to 230 °C at 20 °C increase per min and kept at 230 °C for 13 min.

In the range of temperature studied, FA profiles were rather similar, showing that lipid composition is not very affected by temperature (data not shown). Since yields obtained at 80 °C were lower and the intensity of peaks at 160 °C were slightly lower than those at 120 °C, we selected the intermedia temperature 120 °C as an optimum temperature to obtain *F. vesiculosus* extracts with high lipid content. Figure 3 shows the chromatograms of *F. vesiculosus* PLE extracts obtained at 120 °C with hexane (A), ethyl acetate (B), acetone (C), ethanol (D) and ethanol:water 50:50 (E) and Table 4 shows the FA quantification. In general, the maximum FA content was achieved with ethyl acetate (693.20 mg total FA/g PLE), following by acetone (595.90 mg total FA/g PLE) and ethanol (554.42 mg total FA/g PLE). As expected, results showed that ethanol:water 50:50 (156.23 mg total FA/g PLE) is not a proper solvent for fatty acid extraction from algae and, surprisingly, hexane (426.12 mg total FA/g PLE) did not improve the FA extraction with regards to acetone or ethanol. Comparing the FA profiles obtained with the different solvents, FA proportions were different depending on the solvent used (see Figure 3 and Table 4). For example, ethyl acetate enhanced oleic (C 18:1, peak 8), ARA (C 20:4, peak 10) and EPA (C 20:5, peak 11) with regards to acetone and ethanol since quantities were almost double. However, ethyl acetate did not enhance myristic (C 14:0, peak 2) and palmitic (C 16:0, peak 5) acids. The compounds enhanced correspond with the long chain FAs since they are less soluble in polar solvents than short chain FAs.

Other authors have used SLE and/or PLE with solvents of different polarity for the extraction of FAs from food matrix and microalgae. Castejón and co-workers did not observe significant changes in the composition of chia seed oils extracted with solvents with different polarity [41]. Similarly, da Costa Rodriguez research group studied the effect of solvent composition of rice bran oil extracted with ethanol containing different water contents at 60–90 °C and concluded that there were no major differences in the lipid composition present in the extracts [42]. The use of PLE with green solvents (water and ethanol) for the extraction of bioactives from the microalgae *Nannochloropsis gaditana* and *Phaeodactylum tricornutum* was recently investigated [43,44]. Despite the optimum extraction conditions was achieved with pure ethanol, FAs such as palmitoleic, palmitic, myristic acids and EPA were predominant in all PLE extracts from *Nannochloropsis gaditana* [43]. The PLE of *F. vesiculosus* algae carried out in this work showed that different solvents produced extracts with different FAs content and therefore, the possibility of using PLE as a selective technique for the extraction of specific carbon number fatty acids.

Furthermore, the differences in the FAs content in *F. vesiculosus* PLE extracts have a direct effect on the ω-6/ω-3 ratio. The omega 6 to omega 3 FAs (ω-6/ω-3) ratio is another commonly used index to access healthy diet and thus, good lipid quality [24]. Studies have suggested that a high ω-6/ω-3 ratio in diet is possibly linked to the development of a variety of physiological disorders (such as cancer, coronary heart disease, etc.) [45,46]. Table 4 shows that the ω-6/ω-3 ratios in *F. vesiculosus* PLE extracts are between 2.95 (PLE ethyl acetate) and 1.92 (PLE ethanol:water 50:50) and represent ratios much lower than those recommended by FAO (ω-6/ω-3 = 10) [47]. These good values arise due to *F. vesiculosus* is able to synthetize high EPA content. However, although all fractions showed a low ω-6/ω-3 FA ratio, the better value was obtained for extractions performed with the most polar solvents such as ethanol or ethanol:water 50:50. To summarize, *F. vesiculosus* PLE using ethyl acetate resulted in high lipid yield, improves the extraction efficiency for long chain fatty acids (oleic acid, ARA and EPA) and resulted extract contain lower ω-6/ω-3 fatty acids ratio than the maximum recommended by FAO.

## 3. Material and Methods

### 3.1. Sample Collection and Preparation

Four brown species of marine algae (*F. vesiculosus*, *D. dichotoma*, *C. baccata*, *H. elongata*) and two green algae species (*U. intestinalis*, *U. lactuca*) were freshly collected during the low tide in June 2017 on the Atlantic coast of Galicia (Northwest of Spain). Samples were collected, kept in a polyethylene bags and transported to the laboratory immediately. Afterwards, algae were gently rinsed with distilled water, freeze-dried (LyoBeta 15, Telstar, Terrasa, Spain), grounded (particle size < 500 μm) (Knife Mill Grindomix GM 200, Retsch GmbH, Haan, Germany) and stored four weeks protected from oxygen, light and moisture until use.

### 3.2. Chemicals

FA standards, linoleic acid, γ-linolenic acid, oleic acid, palmitic acid, stearic acid, myristic acid, *cis*-5,8,11,14,17-eicosapentaenoic acid (EPA) and arachidonic acid (ARA) were obtained from Sigma (Madrid, Spain). Ethanol and acetone was obtained from PanReac AppliChem ITW Reagents (Barcelona, Spain). Ethyl acetate, hexane, methanol and chloroform was obtained from Macron Fine Chemicals^TM^ (Gliwice, Poland). All solvents used in this study were high-performance liquid-chromatography or analytical grade, and the water was distilled and processed through an ultrapure water system (Milli-Q Integral 3 Water Purification System, Millipore, Burlington, MA, USA).

### 3.3. Lipid Extraction

Extraction of lipids was done following the method mentioned by Folch et al. [9] and quantified gravimetrically. Briefly, 10 mg of dry weight (DW) algae was soaked in 500 μL of chloroform: methanol (2:1, *v*/*v*) and kept overnight at 4 °C. Next day, algae was sonicated in an ultrasounds bath (Selecta-ultrasons) and mixed by vortex. To ensure complete extraction, another 1250 μL of extraction solvent was added and vortexed. Later, 1300 μL of Milli-Q water was added and the content was vortexed mildly to remove water-soluble impurities. Then, the tubes were centrifuged (4000 rpm, 4 min) for the separation of two layers. Lower lipid phase was transferred carefully to a vial and dried in a fume hood. These dried lipids were then measured gravimetrically.

### 3.4. Pressurized Liquid Extraction (PLE)

Extractions of algae species were performed using an accelerated solvent extractor (ASE, 350, Dionex Corp, Sunnyvale, CA, USA) equipped with a solvent controlled unit. Five different solvents (hexane, ethanol, ethyl acetate, acetone and ethanol 50%) at 3 different temperatures (80 °C, 120 °C, 160 °C) were tested. Extractions were performed in triplicate in 10 mL extractions cells and 100 bar. The amount of 1 g of algae was loaded into the stainless steel cell with sea sand (thin grain, particle size 250–300 μm, Sigma-Aldrich, Madrid, Spain) above and below the sample to avoid any void spaces. Then, the extraction cell was placed into the carrousel and the automatic extraction sequence began with the loading of the cell into the oven. When the cell was heated to the pre-set extraction temperature, the cell was pressurized for 10 min and then allowed to flow the extract into the collection vial. The solvent total volume used was 20 mL.

### 3.5. Fatty Acid Analysis (by GC-MS)

Identification and quantification of FAs were performed by modified method of Miller and Berger [48] as mentioned in Saha et al. [49]. Briefly, a known amount of lipid and PLE samples were saponifed by boiling it with 1 mL of saponification reagent (15 g NaOH in 100 mL of 1:1 methanol: water) for 30 min. The sample was then boiled in a water bath at 80 °C for 20 min with 2 mL of methylation reagent (1:1.18 methanol: 6N HCl). After cooling, 1 mL of extraction solvent (1:1 distilled hexane: anhydrous diethyl ether) was added and mixed thoroughly. Thereafter the lower aqueous phase was discarded and the remaining upper phase was washed with 3 mL of base wash solution (1.2% NaOH *w*/*v*). Finally, one aliquot of the resulting sample was dissolved in hexane to be analyzed by GC-MS. Standards were submitted to the same trans-esterification steps. Fatty acid methyl esters (FAMEs) were analysed by a GC-MS-FID using 7890A System (Agilent Technologies, (Loveland, CO 80537, USA) comprising a split/splitless injector, electronic pressure control G4513A autoinjector, a 5975C triple-axis mass spectrometer detector and GC-MS Solution software. The column used was an Agilent HP-5MS UI capillary column (30 m × 0.250 mm × 0.25 μm). Helium was used as a carried gas at a constant flow of 1.8 mL/min. Oven temperature programme started at 50 °C, increased to 210 °C at 20 °C increase per min and hold for 18 min. Then, temperature was further increased to 230 °C at 20 °C increase per min and kept at 230 °C for 13 min. The injection volume was 1μL in splitless mode. Inlet temperatures was set at 260 °C and MS ion source and interface temperatures were 230 °C and 280 °C respectively. Data were acquired in a full scan from 40 to 500 *m*/*z.*

### 3.6. Antioxidant Activity

The antioxidant activity of the PLE samples was determined by the DPPH scavenging assay based on a procedure described by Brand-Williams et al. [50]. This method consists in the neutralization of free radicals of DPPH (1,1-diphenyl-2-picryl hydrazyl) (Sigma–Aldrich, Spain) by the antioxidant extracts. A dilution series of the extracted samples was prepared (0.25, 0.5, 1, 1.5 mg/mL) and an aliquot (25 μL) of each one was added to 975 μL of DPPH in ethanol (23.5 μg/mL). Reaction was complete after 2 h at room temperature in the dark. Absorbance was then measured at 515 nm against the blank in the Generys 10 uv spectrophotometer reader. Ethanol was used to adjust zero and DPPH–ethanol solution as a reference sample. The DPPH concentration in the reaction medium was calculated from the following calibration curve, determined by linear regression (r = 0.9998): Y = 0.0385X + 0.0083. The percentage of remaining DPPH against the extract concentration was then plotted to obtain the amount of antioxidant necessary to decrease the initial DPPH concentration by 50% or IC_50_. Thus, the lower the IC_50_, the higher the antioxidant power.

### 3.7. Antibacterial Activity

The PLE extracts were individually tested against a Gram-positive, *S. aureus* (ATCC 25923), and Gram-negative, *E. coli* (ATCC 25922), bacterial strains. All tests were performed in Mueller-Hinton broth supplemented with 0.5% tween 20. The inocula of bacterial strains were prepared from overnight Mueller-Hinton broth cultures at 37 °C. Test strains were suspended in Mueller-Hinton broth to give a final density 1 × 10^8^ cfu/mL. The algae extract dilutions in ethanol ranged from 50 to 10 mg/mL. The 96-microwell plates were prepared by dispensing into each well 185 μL of culture broth, 5 μL of the inocula and 10 μL of the different extract dilution. The final volume of each well was 200 μL. Plates were incubated at 37 °C for 24 h for each bacterium. Negative controls were prepared using 10 μL of ethanol, the solvent used to dissolve the algae extracts. Chloramphenicol (Sigma, Madrid, Spain) were used as positive reference standards to determine the sensitivity of the microbial species used. Absorbance was then measured at 620 nm at initial time (t = 0) and at 24 h (t = 24).

### 3.8. Statistical Analysis

Lipid and PLE extraction of algae, fatty acid GC-MS analysis, antioxidant and antibacterial determinations were performed in triplicates. Results are therefore averages of triplicates and the values in figures and tables are shown with the corresponding standard deviations.

## 4. Conclusions

In this study, we assessed the lipid composition, and the antioxidant and antibacterial activities of six macroalgae species from the Northwest of Spain (*F. vesiculosus*, *D. dichotoma*, *C. baccata*, *H. elongata*, *U. intestinalis* and *U. lactuca*). Results showed that *F. vesiculosus* followed by *C. baccata* are the best candidates to exploit them as a nutraceuticals or food products. To better exploit algae potential, there is a need to develop new and enhanced novel extraction technologies without the use of high amounts of toxic organic solvents. We used PLE technology as eco-friendly method to extract high quality lipid from *F. vesiculosus* algae. Effect of the extraction conditions on yield and FA composition of this alga was established. PLE optimization showed that lipid profile was not very influenced by temperature in the range studied (80–160 °C). After testing five different polarity solvents (hexane, ethanol, ethyl acetate, acetone and ethanol:water 50:50), we concluded that ethyl acetate enhances the extraction of long chain fatty acids (oleic acid, ARA and EPA), producing extracts with almost the double in comparison to ethanol and acetone solvents. Although the best (lower) ω-6/ω-3 ratios were achieved with the most polar solvents, such as ethanol and ethanol:water 50:50, all *F. vesiculosus* PLE extracts showed ω-6/ω-3 ratios lower than 3, which are significantly lower than the maximum ratio recommended by FAO (ω-6/ω-3 = 10). Future research priorities in this area should be concentrated on overcoming the challenges of employing this PLE technique on an industrial scale so that the significant benefits to be obtained by improved extraction of FA from *F. vesiculosus* are exploited by industry.

## Figures and Tables

**Table 1:** Lipid content (%) and fatty acid composition (mg/g) of 6 algae species (Dry Weight). Lipids were extracted using the Folch method (*n* = 3). Number of carbon and unsaturation (C:U) status and retention time (RT) of the fatty acid methyl esters (FAMEs) are also included. Results show mean ± standard error of the mean (SEM) of three experiments. N.D means not detected.

	FA (C:U)	RT (min)	*F. vesiculosus*	*C. baccata*	*H. elongata*	*D. dichotoma*	*U. lactuca*	*U. intestinalis*
**FA content (mg/g algae)**	**FA 14:0**	9.802	11.09 ± 0.19	5.13 ± 0.12	1.72 ± 0.04	3.01 ± 0.37	1.78 ± 0.22	1.96 ± 0.23
	**FA 15:0**	10.774	0.31 ± 0.03	N.D	0.17 ± 0.03	N.D	0.18 ± 0.02	0.08 ± 0.00
	**FA 16:1**	11.789	0.98 ± 0.22	2.19 ± 0.04	0.56 ± 0.01	1.16 ± 0.05	0.16 ± 0.00	0.16 ± 0.00
	**FA 16:0**	12.078	9.64 ± 0.30	6.80 ± 0.29	5.85 ± 0.14	4.40 ± 0.64	6.09 ± 0.29	6.02 ± 0.22
	**FA 18:3**	14.900	0.08 ± 0.00	N.D	0.04 ± 0.04	N.D	0.09 ± 0.01	N.D
	**FA 18:2**	15.304	0.34 ± 0.04	0.16 ± 0.02	0.01 ± 0.00	0.01 ± 0.00	0.05 ± 0.01	0.06 ± 0.00
	**FA 18:1**	15.507	13.15 ± 1.03	3.09 ± 0.34	0.49 ± 0.09	1.09±0.05	0.47 ± 0.02	0.23 ± 0.01
	**FA 18:0**	16.041	1.56 ± 0.13	1.65 ± 0.16	1.80 ± 0.04	1.28 ± 0.07	1.68 ± 0.11	2.11 ± 0.08
	**FA 20:4**	20.549	1.30 ± 0.12	0.62 ± 0.01	N.D	N.D	N.D	N.D
	**FA 20:5**	20.806	0.36 ± 0.08	0.24 ± 0.01	N.D	0.15 ± 0.03	N.D	N.D
**FA total (mg/g algae)**			38.83	19.87	10.64	11.09	10.46	10.63
**Lipid content by Folch (%)**			6.6%	6.7%	6.0%	5.7%	4.8%	4.6%

**Table 2:** Antiradical activity of macroalgae ethanolic extracts against DPPH. Results show mean ± standard error of the mean (SEM) of three experiments.

	*Antioxidant Capacity*
	DPPH (IC_50_; μg/mL)
*F. vesiculosus*	7.17 ± 0.01
*D. dichotoma*	>100
*C. baccata*	28.49 ± 3.80
*H. elongata*	64.89 ± 6.64
*U. intestinalis*	>100
*U. lactuca*	>100

**Table 3:** Yields (%) obtained for *F. vesiculosus.* Results show mean ± standard error of the mean (SEM) of three experiments.

*Fucus vesiculosus*					
Extraction Temp.	Yield (%) PLE				
	Hexane	Ethyl Acetate	Acetone	Ethanol	Ethanol:Water 50:50
**80 °C**	2.79 ± 0.12	4.72 ± 0.11	9.01 ± 1.24	8.85 ± 1.51	34.85 ± 3.11
**120 °C**	3.72 ± 0.24	5.59 ± 0.21	10.73 ± 0.23	11.98 ± 1.18	41.49 ± 1.47
**160 °C**	4.49 ± 1.54	7.03 ± 1.79	12.90 ± 1.21	12.89 ± 0.68	57.19 ± 2.38

**Table 4:** Fatty acid composition (mg/g PLE extract) of lipids extracted by PLE from *F. vesiculosus* algae with 5 solvents (hexane, ethyl acetate, acetone, ethanol and ethanol:water 50:50) at 120 °C. Results show mean ± standard error of the mean (SEM) of three experiments. N.D means not detected.

*Fucus vesiculosus*					
FA	FA Quantity (mg/g PLE)				
	Hexane	Ethyl Acetate	Acetone	Ethanol	Ethanol:Water 50:50
FA 14:0	150.51 ± 9.83	227.31 ± 9.43	247.35 ± 5.07	211.62 ± 5.19	29.36 ± 0.52
FA 15:0	2.53 ± 0.15	3.8 ± 0.42	2.97 ± 0.25	2.91 ± 0.11	0.12 ± 0.00
FA 16:1	10.67 ± 0.27	14.21 ± 0.60	14.78 ± 0.56	13.33 ± 0.5	1.89 ± 0.05
FA 16:0	91.37 ± 8.55	150.37 ± 6.01	167.51 ± 7.42	159.16 ± 14.68	84.98 ± 2.04
FA 18:3	0.16 ± 0.27	0.71 ± 0.12	0.39 ± 0.01	0.46 ± 0.07	N.D
FA 18:2	3.76 ± 0.32	6.18 ± 0.17	3.58 ± 0.02	3.47 ± 0.64	0.45 ± 0.02
FA 18:1	131.52 ± 10.54	234.44 ± 6.62	123.19 ± 28.83	126.11 ± 8.48	18.33 ± 0.32
FA 18:0	12.22 ± 1.46	19.75 ± 0.77	16.91 ± 1.21	16.92 ± 1.62	16.43 ± 0.22
FA 20:4	15.65 ± 1.49	23.38 ± 0.82	11.41 ± 0.01	13.02 ± 2.29	2.92 ± 0.04
FA 20:5	6.63 ± 0.66	11.37 ± 0.39	6.94 ± 0.47	7.68 ± 0.58	1.75 ± 0.02
FA 20:3	1.10 ± 0.10	1.68 ± 0.30	0.87 ± 0.05	0.91 ± 0.97	N.D
Total FA	426.12	693.20	595.90	554.42	156.23
Total ω-3	6.63	11.37	6.94	7.68	1.75
Total ω-6	19.57	30.27	15.38	17.43	3.37
ratio ω-6/ω-3	2.95	2.665	2.215	2.208	1.92
